# Measurement of aortic pulse wave velocity in CMR: comparison of transit time estimators

**DOI:** 10.1186/1532-429X-13-S1-P13

**Published:** 2011-02-02

**Authors:** Anas Dogui, Alban Redheuil, Muriel Lefort, Alain Decesare, Alain Herment, Elie Mousseaux

**Affiliations:** 1 Inserm, Paris, France

## Objectives

To investigate the efficacy of a new method (TT-Upslope) for transit time (Δt) estimation from phase contrast CMR flow curves, and to compare it with three previously described methods based on the commonly used foot-to-foot approaches, as well as the point-to-point and wave-to-wave approaches.

## Background

CMR is increasingly used for measuring aortic arch PWV (arch-PWV) by using accurate aortic length and transit time between flow waves. If robust aortic length measurement is an obvious strength of CMR thanks to many 3D imaging approaches available, Δt measurement remains a major challenge. Consequently, different methods have been previously described to estimate the Δt using CMR, but there is to date no fully standardized method for its determination.

## Methods

Fifty healthy subjects underwent carotid-femoral pulse wave velocity (cf-PWV), and carotid pulse pressure (CPP) measurements by applanation tonometry, as well as CMR exams with steady-state free-precession (SSFP) and Phase Contrast (PC) acquisitions at the level of the aortic arch. These data were used for the automated estimation of the arch-PWV, and the aortic areas of the ascending aorta, which were combined with CPP to estimate the local distensibility (aa-Dist). The 3D length of the aortic arch was calculated from axial and coronal SSFP acquisitions. Δt was defined as the time shift between the flow curves of the ascending (CA) and descending (CD) aorta and calculated with: 1) TT-Upslope by minimizing the area delimited by two sigmoid curves fitted to the systolic up-slope of CA and CD, 2) TT-Point using the half maximum of CA and CD, 3) TT-foot using CA and CD feet, 4) TT-Wave by minimizing the area between the whole CA and the CD curves using the cross correlation technique.

## Results

The arch-PWV estimated with both estimators TT-Upslope and TT-Wave resulted in a better correlation with aging, cf-PWV, as well as aa-Dist (Table 1). Furthermore, the TT-Upslope method resulted in a higher reproducibility (4%), a better correlation of arch-PWV with aa-Dist according to the Bramwell-Hill equation, and less overlap between the ≤37 years (n=25) and ≥38 years (n=25) age groups.

**Table1:** Pearson coefficients of the regression analysis between arch-PWV and age, cf-PWV, aa-Dist

	*Linear regression*	*Power regression*	
Δt estimators	Age/arch-PWV	cf-PWV/arch-PWV	arch-PWV/aa-Dist
TT-Wave	r=0.83 p<0.001	r=0.7 p<0.001	r=0.71 p<0.001
TT-Upslope	r=0.83 p<0.001	r=0.69 p<0.001	r=0.81 p<0.001
TT-Foot	r=0.47 p<0.001	r=0.34 p<0.013	r=0.61 p<0.001
TT-Point	r=0.72 p<0.001	r=0.59 p<0.001	r=0.6 p<0.001

## Conclusions

TT-Upslope and TT-Wave appear to be less sensitive to the low temporal resolution, signal-to-noise ratio, and varying profile of velocity curves. Indeed, they avoid the restriction of the analysis to a few points of the velocity curve, and provide better correlations with the physiological and stiffness parameters.

